# Sex-dependent transcriptional responses and druggable targets in gentamicin-induced nephrotoxicity

**DOI:** 10.1007/s00210-026-04997-4

**Published:** 2026-01-24

**Authors:** Pınar Portakal, Tuğba Gürkök-Tan

**Affiliations:** Food and Agriculture Vocational School, Cankiri Karatekin University, Çankırı, Turkey

**Keywords:** Drug–gene interactions, Gentamicin nephrotoxicity, Hub genes, Network analysis, Sex differences, Transcriptomics

## Abstract

Gentamicin is a potent aminoglycoside antibiotic, but its clinical use is limited by severe nephrotoxicity. Experimental and transcriptomic studies have indicated that the extent of renal injury and recovery following gentamicin exposure may differ between sexes, yet the underlying molecular mechanisms remain poorly understood. In this study, we investigated sex-specific transcriptional signatures, hub genes, transcription factors, and druggable molecular pathways involved in gentamicin-induced kidney injury and recovery. RNA-seq data from rat kidney tissues (male, female, and combined groups) were obtained from the NCBI-GEO database and analyzed at two time points corresponding to injury (day 15) and recovery (day 29). Differentially expressed genes were identified and subjected to functional enrichment (GO/KEGG), hub gene, and transcription factor detection analyses, followed by drug–gene interaction screening using DGIdb. Males exhibited a broader but more homogeneous transcriptional response, whereas females showed a narrower yet more pronounced activation pattern. A conserved mitotic core (*BIRC5*, *BUB1B*, *CCNA2*, *CDC20*) was shared across sexes and phases. Sex-dependent divergence emerged primarily during the recovery phase: *PBK*, *CCNB1*, and *NUF2* were identified as female-specific proliferative hubs, whereas CDCA8 was uniquely enriched in males. Transcription factors *CENPA*, *FOXM1*, and *MYBL2* formed a shared regulatory backbone, while *DNMT1* appeared predominantly in male injury samples, suggesting a male-biased epigenetic component. DGIdb integration showed that among sex-specific hub genes, only CCNB1 had druggable interactions, whereas PBK, NUF2, and CDCA8 lacked pharmacological matches. In contrast, several core cell-cycle regulators (BIRC5, BUB1B, BUB1, CCNA2, CDK1) were highly druggable. These findings demonstrate that gentamicin nephrotoxicity is governed by a shared cell-cycle core but modulated by sex-specific repair programs, providing a molecular basis for the development of sex-tailored therapeutic strategies.

**Supplementary Information:**

The online version contains supplementary material available at 10.1007/s00210-026-04997-4.

## Introduction

Aminoglycoside antibiotics, particularly gentamicin, have been widely used in clinical treatments for many years due to their potent bactericidal activity against Gram-negative bacteria. However, the most clinically significant side effect of gentamicin is nephrotoxicity, which develops depending on the dose and duration of treatment. Gentamicin tends to accumulate in the renal cortex and has a toxicity profile characterized by lysosomal dysfunction, mitochondrial damage, oxidative stress, and apoptotic/necrotic cell death, particularly in proximal tubule cells (Ali et al. 2011). Clinical studies show that approximately 10–20% of patients receiving aminoglycosides develop renal dysfunction (Lopez-Novoa et al. 2011; Harvey and Alvarez De La Rosa 2025). Despite their antibacterial activity, this situation severely limits the therapeutic use of aminoglycosides.

In recent years, it has been increasingly recognized that biological sex is a determining variable in the development and recovery from kidney injury. Experimental and clinical observations indicate that males are more susceptible to gentamicin-induced nephrotoxicity, while females are partially protected (Neugarten 2020; Stamellou et al. 2024). This difference is driven by the antioxidant and immunomodulatory effects of estrogen, as well as the potentiation of prooxidative and inflammatory responses by androgens (Kumar and Brooks 2023). Estrogen enhances antiapoptotic signaling (e.g., via the PI3K/Akt pathway) and transcriptional activation of antioxidant genes, while testosterone increases ROS production and inflammatory cytokine expression (Conte et al. 2023; Harvey and Alvarez De La Rosa (2025).

Bioinformatics analyses are a powerful approach to filling this knowledge gap by integrating large-scale omics data. The combined use of methods such as differential gene expression, protein–protein interaction networks, transcription factor analyses, and drug-target mappings provides a systems-level understanding of complex biological responses (Subramanian et al. 2005; Kanehisa et al. 2023). This strategy not only deepens mechanistic understanding but also offers opportunities for drug repositioning.

In recent years, sex-specific gene expression has become an increasingly important area of research in renal pathophysiology. RNA-seq–based studies have revealed that processes such as the cell cycle, DNA repair, immune response, and metabolic adaptation in kidney tissue are differentially regulated by sex (Chen et al. 2025; Si et al. 2009). However, the vast majority of existing transcriptomic studies on gentamicin nephrotoxicity have not considered sex as an independent biological factor and have not conducted a comprehensive examination of transcription factors, hub gene networks, and potential pharmacological targets.

Qiu et al. (2014), one of the pioneering studies in this field, conducted a transcriptomic time series analysis covering the time points D2, D4, D8, D15, and recovery day (R29) in rat kidney tissues following gentamicin administration and identified general gene expression patterns during this period. However, although samples were separated by sex in the RNA-seq dataset obtained in that study, sex-specific transcriptional differences were only reported superficially; transcription factor-hub gene relationships or gene network dynamics were not examined in detail.

In this study, the dataset created by Qiu et al. (2014) was reanalyzed, and sex-specific transcriptomic differences in gentamicin nephrotoxicity were assessed at the systemic level for the first time. Furthermore, by selecting the injury phase (day 15) and recovery phase (day 29) from among multiple time points included in the original study, time- and sex-dependent gene expression changes, hub gene networks, transcription factor profiles, and DIGDB-based pharmacological target mappings were examined in both male and female samples using an integrative bioinformatics approach. This methodological approach goes beyond previous analyses by revealing the sex-based dynamics of the molecular repair process in gentamicin nephrotoxicity.

The hypothesis proposed in this direction is that the sex-related differences observed in gentamicin nephrotoxicity are due to the cell cycle, epigenetic reprogramming, and proliferative repair processes that are shaped by regulatory gene networks, and specific genes within these networks form pharmacologically modulatable molecular nodes. Thus, the data obtained are expected to provide translational contributions to the development of sex-sensitive treatment approaches for aminoglycoside-induced kidney injury.

## Materials and methods

### RNA-seq data source and experimental design

Publicly available RNA-seq datasets were obtained from the NCBI Gene Expression Omnibus (GEO) database under accession number GSE50804, originally published by Qiu et al. (2014) (time-series pattern of gene expression profile in gentamycin-induced nephrotoxicity). This dataset includes renal cortical tissue samples from male and female Sprague–Dawley rats intramuscularly administered gentamicin sulfate (80 mg/kg/day) or saline as control. Animals were sacrificed at five time points corresponding to the progression and recovery of nephrotoxicity: day 2, day 4, day 8, day 15, and recovery day 29, with three biological replicates for each condition. The experiments were conducted using the Agilent-014879 Whole Rat Genome Microarray 4 × 44 K G4131F platform.

In the present study, we re-analyzed this dataset with a focus on sex-dependent molecular responses during two key phases of the gentamicin nephrotoxicity model: the injury phase (day 15) and the recovery phase (day 29). A total of 24 RNA-seq libraries were included in the analysis, representing male and female samples at these two time points and their respective saline controls. Sample identifiers and metadata (GSM accession numbers, treatment conditions, sex, and replicates) are provided in Supplementary Table S1.

### Data preprocessing and differential gene expression analysis

Differentially expressed genes (DEGs) were identified using publicly available RNA-seq data (GSE50804) through the GEO2R web interface, which implements the limma (Linear Models for Microarray and RNA-seq Data) statistical framework. Exploratory data visualization and quality control were initially performed using the GEO2R platform, which provides built-in graphical tools for evaluating data consistency and normalization. Principal component analysis (PCA), scatter plots, and box plots generated via GEO2R were used to assess overall expression distribution, identify potential outliers, and verify clustering of biological replicates across experimental groups (D15 and D29, male and female). Following data inspection, normalized expression matrices were imported into R (v4.3.1) for batch effect correction. The sva package (ComBat function) was used to adjust for possible inter-sample variability unrelated to biological factors. The effectiveness of batch correction was confirmed by visual re-evaluation of sample clustering through PCA and box plot inspection.

Genes exhibiting an absolute log₂ fold change (|log₂FC|) ≥ 1 and a false discovery rate (FDR) < 0.05 (Benjamini–Hochberg correction) were considered significantly differentially expressed. All analyses were conducted separately for two time points—the injury phase (day 15) and the recovery phase (day 29) and across three biological subgroups: combined (general), male, and female. This stratified design enabled the identification of both time-specific and sex-dependent transcriptional alterations associated with gentamicin-induced nephrotoxicity.

### Functional enrichment analysis (GO and KEGG)

The resulting differential gene lists were subjected to Gene Ontology (GO) and Kyoto Encyclopedia of Genes and Genomes (KEGG) enrichment analysis using ClusterProfiler and DAVID. A significance threshold of FDR < 0.05 was used in analyses conducted in the biological process (BP), molecular function (MF), and cellular component (CC) categories. Enriched pathways and processes were compared by sex subgroups, and common and different pathways were presented with Venn diagrams.

### Protein–protein interaction (PPI) network, hub gene, and transcription factor (TF) analysis

The interaction network of differentially expressed genes was constructed using the STRING v11.5 database (http://string-db.org/). Genes integrated into the network were visualized in Cytoscape (v3.9.1), and genes with the highest linkage scores were identified using the MCODE and Maximal Clique Centrality (MCC) algorithms. The 10 highest-scoring hub genes were selected for each subgroup. Potential transcriptional regulators of hub genes were identified using the ChEA3 (ChIP-X Enrichment Analysis 3) platform (Keenan et al. 2019).

### Pharmacological targeting and repositioning analysis

Hub genes obtained from sex-stratified PPI analyses for the injury and repair phases were screened for drug–gene interactions using the DGIdb (v5.0.10) platform. Interaction types involving inhibitors, antagonists, and antibodies were prioritized based on DGIdb’s predefined multi-source interaction definitions. An interaction score of ≥ 1.0 was applied as a reliability threshold. The strongest match for each gene was determined by considering the interaction score, regulatory approval (approved/not approved), interaction direction (type and directionality), and, if applicable, mechanism of action fields. In sex-specific analyses, hub genes that occurred only in one sex and one phase (D29–D15–female: PBK, CCNB1, NUF2; D29–D15–male: CDCA8) were evaluated in separate sub-analyses using the same filtering strategy, and the results were reported preserving the phase-sex distinction.

## Result

### Gene expression profiles and sex-specific differences in gentamicin-induced nephrotoxicity

Bioinformatic analysis revealed that gentamicin exposure resulted in significant transcriptional reprogramming, with these changes exhibiting sex-specific differences in both the injury (D15) and recovery (D29) phases. GEO2R plots confirmed that sample distributions were comparable across groups and exhibited only minimal batch-associated variation (batch effect < 1). PCA analysis showed strong sample clustering, clearly separating phases and sexes. Box plot evaluations demonstrated uniform expression ranges and successful normalization across replicates. Volcano plots provided a clear overview of significantly up- and downregulated genes for each comparison (Supplementary Fig. 1).

**Fig. 1:**
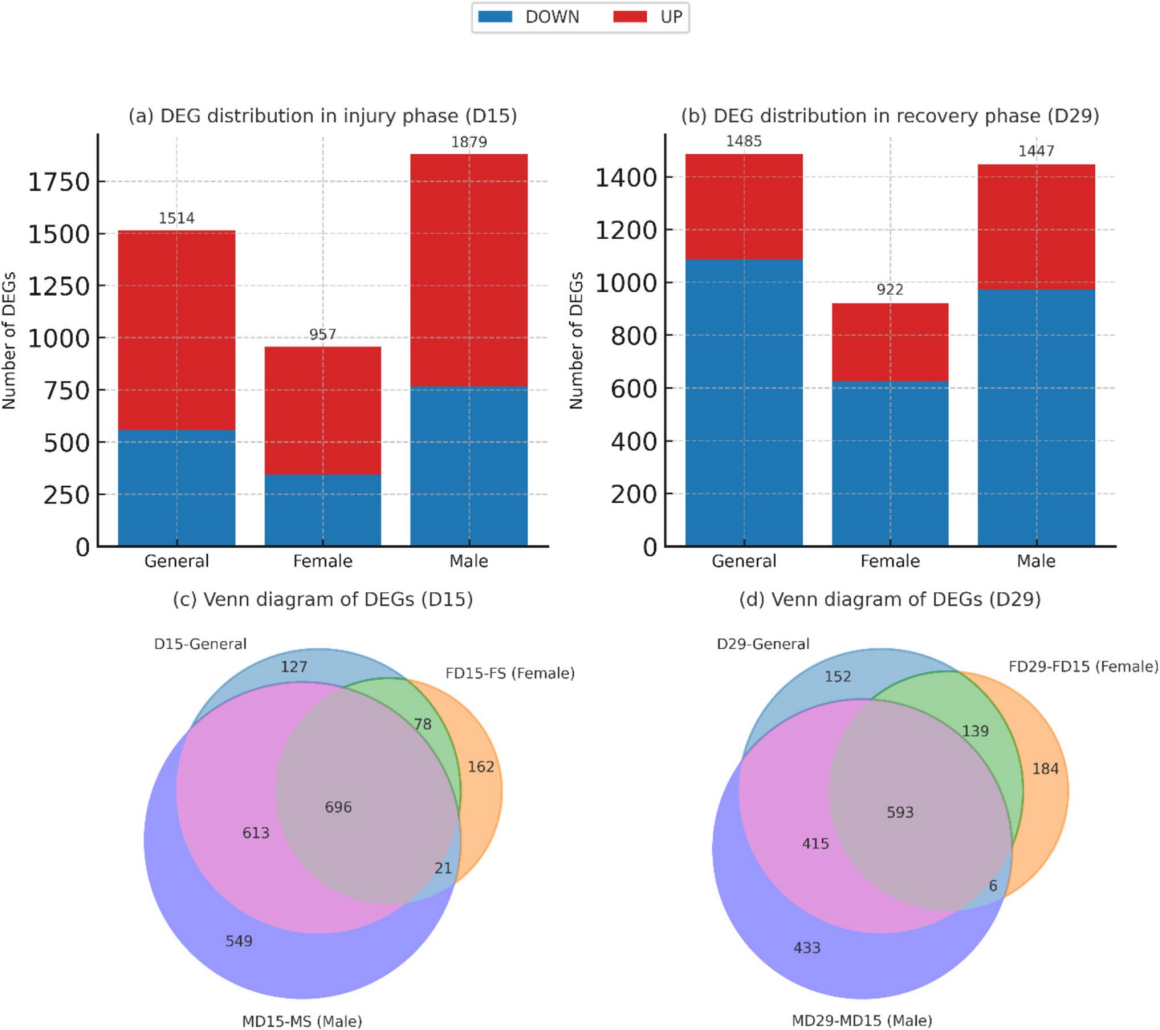
Distribution of differentially expressed genes (DEGs) in gentamicin-induced nephrotoxicity. **a** Distribution of up- and downregulated genes in the injury phase (D15) across general, female, and male groups. **b** Distribution of up- and downregulated genes in the recovery phase (D29) across general, female, and male groups. **c** Venn diagram showing overlapping and unique DEGs across groups in the injury phase (D15). **d** Venn diagram showing overlapping and unique DEGs across groups in the recovery phase (D29). The bar plots quantitatively summarize sex-dependent transcriptional differences, while the Venn diagrams highlight both conserved and sex-specific gene expression patterns. Shared DEG clusters primarily reflect common inflammatory and innate immune responses to gentamicin exposure, whereas sex-specific gene sets suggest differential regulation of cell cycle control, immune signaling, and metabolic adaptation, as further supported by functional enrichment analyses

Genes with both general and sex-specific differential expression were identified during the injury and recovery phases (Supplementary File 2). A total of 1879 DEGs (1112 upregulated, 767 downregulated) were identified in males during the injury phase, and 957 DEGs (615 upregulated, 342 downregulated) were identified in females. A combined analysis identified 1514 DEGs (958 upregulated, 556 downregulated), suggesting that these genes represent a common response core between the sexes (Fig. 1a). This distribution suggests that the transcriptional response in males is more widespread but relatively homogeneous, while in females, it is more limited but exhibits a distinct and intensely focused response pattern due to the sharper distribution of DEGs. A general downregulation trend was observed during the recovery phase. In the combined analysis, a total of 1485 DEGs (1086 downregulated, 399 upregulated) were identified, with 922 DEGs (626 downregulated, 296 upregulated) in the female group and 1447 DEGs (973 downregulated, 474 upregulated) in the male group (Fig. 1b). This distribution suggests that transcriptional activity is generally suppressed during the healing phase, and a silencing molecular response is prominent. During the injury phase, 696 common DEGs were identified between the male and female groups; in addition, 549 DEGs specific to males and 162 DEGs specific to females were identified (Fig. 1c). This suggests that while a broad core of common responses exists during the injury phase, both sexes have developed unique adaptive gene sets. During the healing phase, 593 common DEGs were conserved, 433 DEGs specific to males, and 184 DEGs specific to females were identified (Fig. 1d). These findings suggest that common regulatory systems persist during the healing process, but repair mechanisms are differentially supported by sex-specific genes.

A significant number of genes commonly regulated in male and female groups were identified in both phases. Significant differential expression was observed in 696 DEGs in the injury phase and 593 DEGs in the recovery phase across both male and female analyses. These gene clusters suggest that the fundamental cellular response to gentamicin nephrotoxicity is driven by a central set of genes independent of sex, and that this central response becomes more selective during the repair process. In conclusion, despite sex differences in expression, these conserved gene networks represent critical molecular mechanisms regulating the transition between phases of kidney injury and recovery.

To provide deeper mechanistic insight into sex-dependent dimorphism in gentamicin-induced nephrotoxicity, a targeted panel of differentially expressed genes was extracted from the DEG list and systematically evaluated based on their transcriptional behavior and biological relevance (Table 1). Genes were selected according to their established involvement in sex hormone signaling pathways (estrogen, androgen, and glucocorticoid), immune and inflammatory regulation, epigenetic modulation, metabolic homeostasis, and cell-cycle–associated tissue repair. This integrative approach enabled a quantitative comparison of injury- and recovery-phase transcriptional responses between female and male kidneys without imposing a priori sex-biased classifications. Across this gene panel, the majority of cell-cycle–associated genes (*FOXM1*, *MYBL2*, *CENPA*, *CCNB1*, *CCNA2*, *CDK1*, *BUB1*, *BUB1B*, *CDCA8*, *CENPF*) exhibited increased expression during the injury phase in both sexes, followed by reduced expression during the recovery phase. Notably, the extent of repression during recovery was greater in males for *CENPA* and *CCNA2*.

**Table 1:** Sex-dependent transcriptional responses of selected genes during gentamicin-induced nephrotoxicity

Functional category	Gene symbol	Injury female	Injury male	Recovery female	Recovery male
Estrogen/cell cycle	*FOXM1*	+ 1.46	+ 1.65	− 2.03	− 2.45
	*MYBL2*	+ 2.51	+ 2.73	− 3.25	− 3.88
	*CENPA*	+ 2.14	+ 2.42	− 3.22	− 4.18
	*CCNB1*	+ 2.24	+ 2.69	− 4.81	− 4.75
	*CCNA2*	+ 3.24	+ 3.04	− 3.88	− 4.38
	*CDK1*	+ 2.51	+ 2.55	− 5.21	− 4.83
	*BUB1*	+ 2.27	+ 2.36	− 3.61	− 3.64
	*BUB1B*	+ 3.23	+ 2.66	− 5.55	− 5.34
Estrogen–NF-κB	*IL1RN*	+ 1.96	+ 3.42	− 2.12	− 1.20
	*SOCS3*	+ 1.66	+ 1.53	− 1.44	− 1.47
Epigenetic regulation	*DNMT1*	NA	+ 1.37	NA	− 1.20
	*NR3C1*	NA	− 1.40	NA	+ 1.51
Immune response	*TLR2*	NA	+ 1.46	NA	NA
	*TLR4*	+ 1.20	+ 2.02	− 1.27	− 1.90
Chemokine	*CCL2*	+ 2.58	+ 2.83	− 1.63	− 1.38
	*CXCL10*	+ 3.11	+ 3.42	− 2.62	− 2.95
Metabolism	*ACADM*	NA	− 1.00	NA	NA
	*HADHA*	NA	− 1.17	NA	+ 1.52
	*HADHB*	NA	− 1.28	NA	NA
Cell cycle	*CDCA8*	+ 1.94	+ 2.20	− 1.75	− 1.95
	*CENPF*	+ 2.52	+ 2.85	− 4.73	− 5.18

Genes involved in immune and inflammatory signaling (*TLR2*, *TLR4*, *CCL2*, *CXCL10*) showed stronger induction during injury in males compared with females. In parallel, epigenetic and stress-related regulators (*DNMT1*, *NR3C1*) and metabolic genes (*ACADM*, *HADHA*, *HADHB*) displayed predominantly male-specific differential expression patterns.

### Enrichment profiles of GO terms and KEGG pathways in gentamicin nephrotoxicity

**Fig. 2:**
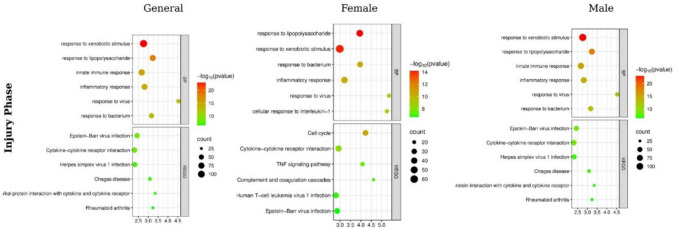
GO biological processes (BP) and KEGG pathway enrichment profiles in the injury phase (D15) across general, female, and male groups. **a** BP enrichment results for the general group. **b** BP enrichment results for the female group. **c** BP enrichment results for the male group. **d** KEGG pathway enrichment for the general group. **e** KEGG pathway enrichment for the female group. **f** KEGG pathway enrichment for the male group. Dot plots represent enriched terms ranked by statistical significance (–log10 *p*-value), gene counts, and category-specific clustering patterns. While inflammatory and innate immune pathways are commonly enriched across all groups, sex-specific differences are evident. Female samples show enrichment of pathways related to cell cycle regulation and proliferative responses, whereas male samples display a predominance of immune–inflammatory signaling and metabolic stress-related pathways, underscoring distinct sex-dependent physiological responses during gentamicin-induced kidney injury

When the enrichment analyses for the injury phase are evaluated together, a common and strong inflammatory-antiviral response core is observed across all groups (Fig. 2). In the GO-BP results, response to xenobiotic stimuli, response to lipopolysaccharide, response to bacteria, inflammatory/immune response, and viral defense processes were ranked highest in all three analyses. Similarly, in the KEGG analysis, viral infection pathways (Epstein-Barr virus infection, Herpes simplex virus 1 infection), cytokine-cytokine receptor interaction, TNF signaling pathway, complement and coagulation cascades, and antigen processing and presentation were enriched in all three groups (Supplementary File 3). This match demonstrates that a strong and common innate immune activation underlies gentamicin-induced kidney damage.

Sex-specific differences emerged through processes superimposed on this core response. In the female group, in addition to immune response terms, cell cycle and proliferation-related processes such as the mitotic spindle checkpoint, cell division, chromosome segregation, and spindle organization were ranked higher in both GO and KEGG analyses (Supplementary File 3). In the male group, while immune/inflammatory processes were preserved, terms related to metabolic and stress adaptation, such as glucocorticoid response, ethanol response, fatty acid β-oxidation, xenobiotic metabolism, and T cell proliferation, were more significantly enriched. Thus, while a common inflammatory-antiviral response predominated in all groups during the injury period, this response was complemented by cell cycle reprogramming in females and metabolic and stress response processes in males.

**Fig. 3:**
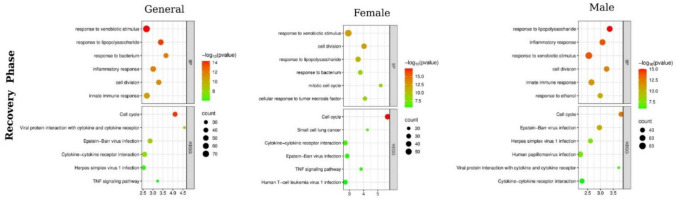
GO biological processes (BP) and KEGG pathway enrichment profiles in the recovery phase (D29) across general, female, and male groups. **a** BP enrichment results for the general group. **b** BP enrichment results for the female group. **c** BP enrichment results for the male group. **d** KEGG pathway enrichment for the general group. **e** KEGG pathway enrichment for the female group. **f** KEGG pathway enrichment for the male group. The recovery phase is characterized by a dominant proliferation- and cell cycle–oriented repair program in females, whereas males exhibit persistent immune–inflammatory signaling, highlighting sex-specific physiological recovery strategies consistent with the transcriptomic patterns observed in Fig. 1

In the overall GO-BP analysis of the recovery phase (Fig. 3), cell cycle, cell division, G1/S transition, chromosome and sister chromatid segregation, DNA replication, and mitotic processes were ranked highest. This biological pattern coincides with the enrichment of cell cycle, DNA replication, PI3K–Akt signaling, and cytoskeleton–motor protein pathways in the KEGG analysis. Furthermore, although chemokine signaling and viral defence terms were not entirely absent, it was clearly observed that the recovery phase was predominantly characterized by proliferation, chromosomal rearrangement, and structural/functional tissue repair processes.

When sex was considered, the pattern of the recovery response differed significantly. In the female group, GO results strongly indicated proliferation-repair-related processes such as mitotic functions, mitotic spindle organization, DNA replication initiation, sister chromatid segregation, DNA repair, and interleukin-1 response. KEGG analysis supported this structure with enrichment in cell cycle, DNA replication, p53 signaling, oocyte meiosis, homologous recombination, and cellular senescence pathways. In the male group, inflammatory/immune response processes (LPS response, inflammation, viral defense, interleukin-1 response, TNF production), stress/metabolic response terms, and glucocorticoid response remained at the top of the rankings in the GO-BP analysis during the recovery phase. Conversely, KEGG analysis showed enrichment in viral infection pathways, cytokine–cytokine receptor interaction, Toll-like/NOD-like receptor signaling, chemokine signaling, and phagosome pathways. This suggests that females tend toward a distinct proliferative-repair profile during the recovery phase, while males maintain a partial dominance of immune/inflammatory response components.

**Fig. 4:**
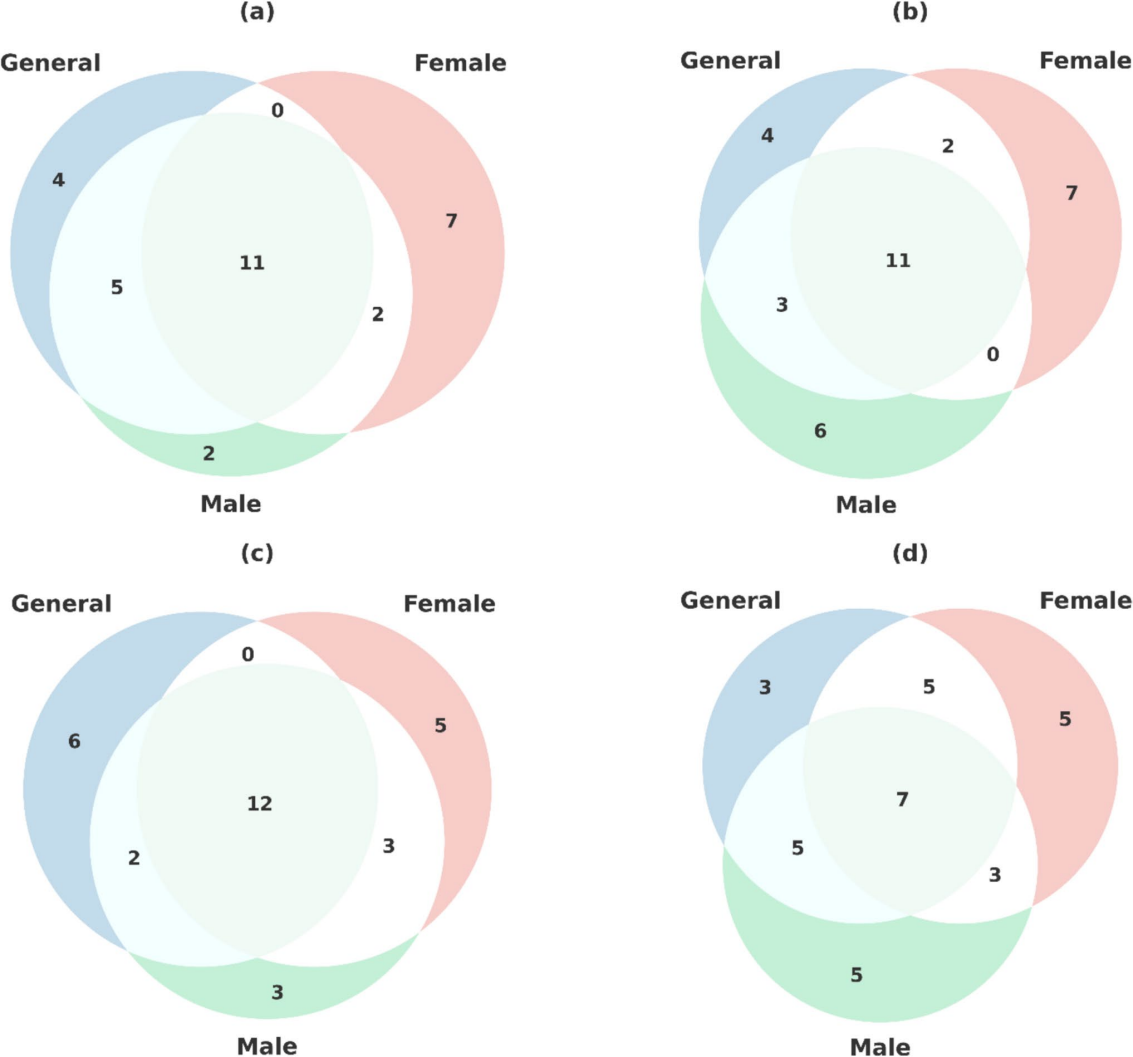
Overlap of enriched biological processes and pathways among sex-specific groups in the injury (D15) and recovery (D29) phases of gentamicin-induced nephrotoxicity. **a** GO biological processes in the injury phase (D15). **b** GO biological processes in the recovery phase (D29). **c** KEGG pathways in the injury phase (D15). **d** KEGG pathways in the recovery phase (D29). Numbers in the Venn diagrams represent the distribution of significantly enriched terms (FDR < 0.05) across the combined (general), female (female), and male (male) subgroups. Overlapping regions indicate a conserved core biological response to gentamicin exposure, whereas non-overlapping regions reflect sex-specific enrichment patterns, suggesting divergent regulatory programs governing injury and recovery processes between females and males

The Venn diagram (Fig. 4) demonstrates that a distinct core of biological responses is common to both the injury and recovery phases of gentamicin nephrotoxicity, but the processes surrounding this common pathway exhibit sex-specific differences. During the injury phase, a high number of common terms shared by the three groups in both GO and KEGG analyses indicate a strong and unified inflammatory-antiviral defense response, while processes unique to female and male subgroups (e.g., cell cycle in women and metabolic-stress response in men) are added in a limited but distinctive way. In the recovery phase, the number of common terms appears to have decreased relatively, while the number of terms specific to each subgroup has increased. This shift suggests that the homogeneous inflammatory response during the injury phase has been replaced by more differentiated, sex-differentiated recovery mechanisms during the recovery phase. At the GO level, women exhibit a recovery model focused on proliferation and cell cycle-repair, while men exhibit a recovery model that preserves immune/inflammatory components for a longer period. At the KEGG level, the decrease in common pathways during the recovery phase suggests that the restructuring process proceeds through gender-specific biological modules. These results suggest that although recovery after gentamicin injury is mediated by the same biological core in all groups, the pattern of recovery exhibits a different organization by sex.

### Identification and functional significance of sex- and phase-specific hub genes and transcription factors

PPI network analysis revealed that hub genes associated with cell cycle and mitosis play a central role in both the injury and recovery phases. During the injury phase, *Cdk1*, *Bub1b*, *Ccna2*, *Cdc20*, *Birc5*, *Cenpf*, *Dlgap5*, *Kif20a,* and *Kif2c* genes were identified as common in all three groups (Table 2, Supplementary File 4). In this phase, *Bub1* was identified only in females, while *Nusap1* was identified in males and in general. *Nusap1* was significantly detected in the general group due to its high expression in male samples, demonstrating a male-dominant regulatory signature during the injury phase. In the recovery phase, *Birc5*, *Bub1*, *Bub1b*, *Ccna2*, *Cdc20*, and *Dlgap5* were identified as common hub genes conserved across groups. In contrast, *Ccnb1*, *Nuf2*, and *Pbk* were identified only in the female group; *Cdca8* and *Cenpf* were identified only in the male group (Table 2).

**Table 2:** Table summarizes the top hub genes and the leading transcription factors across injury (D15) and recovery (D29) phases in the general and sex specific

Phase	Sex	Hub genes (MCC)	TFs (ChEA3 top)
Injury	General	Kif20a, Cenpf, Cdk1, Bub1b, Ccna2, Cdc20, Nusap1, Kif2c, Birc5, Dlgap5	CENPA, FOXM1, MYBL2, DNMT1
	Female	Ccna2, Birc5, Dlgap5, Kif2c, Bub1b, Bub1, Kif20a, Cdc20, Cdk1, Cenpf	CENPA, FOXM1, MYBL2
	Male	Ccna2, Kif2c, Dlgap5, Nusap1, Kif20a, Cenpf, Cdk1, Birc5, Bub1b, Cdc20	CENPA, FOXM1, MYBL2, DNMT1
Recovery	General	Cdk1, Dlgap5, Cdc20, Bub1b, Birc5, Ccna2, Kif2c, Bub1, Kif20a, Nusap1	CENPA, FOXM1, MYBL2
	Female	Pbk, Birc5, Bub1b, Cdc20, Kif2c, Ccna2, Ccnb1, Dlgap5, Nuf2, Bub1	CENPA, FOXM1, MYBL2
	Male	Bub1b, Cdc20, Cdk1, Dlgap5, Cdca8, Birc5, Ccna2, Kif20a, Cenpf, Bub1	CENPA, FOXM1, MYBL2, DNMT1

These findings suggest that a sex-independent core hub gene set (e.g., *Birc5*, *Bub1b*, *Ccna2*, *Cdc20*) persists throughout both the injury and recovery phases of gentamicin-induced nephrotoxicity. However, female-specific proliferative genes (e.g., *Ccnb1*, *Nuf2*, *Pbk*) appear to be more involved in DNA replication and mitotic spindle organization, while male-specific hub genes (e.g., *Cdca8*) are associated with chromosome segregation, cell cycle checkpoints, and epigenetic regulation.

In light of these data, the kidney tissue response to gentamicin injury is shaped around a common proliferative axis driven by core cell cycle genes, while the recovery process proceeds through distinct sex-dependent regulators. This suggests that the speed and effectiveness of repair are mediated by sex-based molecular strategies.

*ChEA3*-based analyses showed that the transcription factors *CENPA*, *FOXM1*, and *MYBL2* were ranked highest in all comparison groups (Table 2, Supplementary File 4). Each of these three factors showed complete overlap with 10 hub genes, indicating that the transcriptional backbone governing cell cycle and mitosis is preserved in both the injury and recovery phases. As shown in Table 2, *CENPA* was identified as the most strongly associated TF in all groups, with the lowest mean rank values (range 2–5). *FOXM1* was ranked second (approximately 5–6 mean rank), while *MYBL2* stood out as a relatively weaker but persistent transcription factor with higher mean rank values (9–12). In contrast, *DNMT1* was identified only in the male groups and in the injury phase overall analysis, with relatively high mean rank values (9.5–15.5). This pattern indicates that the epigenetic contribution of DNMT1 is male-biased, suggesting that the transcriptional response to gentamicin nephrotoxicity is not limited to cell-cycle regulation but also involves a distinct epigenetic reprogramming component that is particularly prominent in males.

### Drug-gene interaction DGIdb

A DGIdb-based retargeting analysis identified 14 high-confidence drug-gene interactions with an interaction score ≥ 1.0 (Table 3). Most of the interactions were inhibitory, with only lubiprostone and niflumic acid classified as activators. Specifically, the experimental inhibitors identified for *BUB1B*, *BUB1*, *CCNA2*, *CCNB1*, *CDK1*, and *BIRC5* suggest that the mitotic programs disrupted in gentamicin nephrotoxicity can be pharmacologically targeted.

**Table 3:** Candidate drugs targeting hub genes in gentamicin-induced kidney injury

Gene	Candidate drug	Interaction type	Interaction score	Approval
BUB1B	3-Phenyl-CPP	Inhibitor	8.70	Experimental
BUB1	GATX2	Inhibitor	6.52	Experimental
CCNA2	Cordycepin	–	6.52	Experimental
BUB1B	DIDS	Inhibitor	3.48	Experimental
BUB1	Lubiprostone	Activator	3.26	Approved
CCNA2	IGN523	Antibody (inhibitory)	3.26	Experimental
CCNB1	Seliciclib	CDK inhibitor	2.32	Experimental
BUB1	Diphenylamine-2-carboxylic acid	Inhibitor	2.17	Experimental
CDK1	Aruncin B	–	2.08	Experimental
BUB1B	Niflumic acid	Activator	1.33	Approved/NSAID
CCNB1	Protoapigenone	–	1.24	Experimental
BIRC5	LY2181308	Inhibitor	1.18	Experimental
BIRC5	Plevitrexed	Inhibitor	1.18	Experimental
CDK1	Protuboxepin A	–	1.04	Experimental

## Discussion

It is known that there are sex-based differences in the response to kidney injury, but existing transcriptional studies have not comprehensively elucidated the gene regulatory networks underlying these differences. Specifically, in gentamicin-induced nephrotoxicity models, sex-specific transcriptional responses have not been systematically assessed at the hub gene and transcription factor level. In this study, gene expression changes observed during the injury and recovery phases of gentamicin-induced nephrotoxicity in rats were evaluated within the context of sex, and potential molecular differences were analyzed using bioinformatics methods. In this context, our study re-evaluates RNA-seq data, previously analyzed only at the time-series level, revealing both the injury and recovery phases and sex-specific molecular responses using an integrative systems biology approach.

### Phase- and sex-dependent transcriptional regulation patterns

Following gentamicin administration, males exhibited more extensive transcriptional reprogramming during the injury phase (1879 DEGs), while females exhibited a more limited but focused response (957 DEGs). During the recovery phase, a predominant downregulation pattern was noted in both sexes (females: 922; males: 1447), suggesting that global transcriptional repression is prominent during the repair phase. This pattern is consistent with the classical timeline of aminoglycoside nephrotoxicity, characterized by acute injury → proliferative compensation → transcriptional dampening/reorganization, and is consistent with the pathogenesis of oxidative stress, inflammation, and mitochondrial dysregulation highlighted in previous experimental reviews (Lopez-Novoa et al. 2011; Quirós et al. 2015).

These findings suggest that gentamicin-induced nephrotoxicity leads to extensive transcriptional reprogramming during both the injury and recovery phases and that sex-specific molecular mechanisms become evident during this process. The high number of differentially expressed genes in the injury phase reflects the early processes of oxidative stress, mitochondrial dysfunction, and proliferative activation in renal proximal tubule cells (Gerhardt et al. 2021). In contrast, the significant downregulation trend observed during the recovery phase is consistent with the repression of cell cycle genes during the repair process and the return of transcriptional activity to physiological levels (Jia et al. 2022).

Venn diagrams indicate a conserved response core shared by both sexes, represented by 696 common genes in the injury phase and 593 in the recovery phase. In contrast, male- and female-specific clusters suggest that the response is shaped by sex-specific modules. Indeed, the literature has reported that males are more likely to develop severe damage to gentamicin, while more acute inflammatory responses predominate in females (Viñas et al. 2020); our findings confirm this differentiation at a stage-based transcriptomic scale.

### Sex-specific functional response profiles

Gentamicin is one of the compounds with the strongest nephrotoxic potential among the aminoglycoside antibiotics, exerting its toxic effects primarily through mitochondrial dysfunction, reactive oxygen species (ROS) production, and lysosomal damage in proximal tubule cells (Lopez-Novoa et al. 2011). In this process, there is a strong pharmacological interaction between cell cycle regulation, inflammatory signaling, and apoptosis. GO and KEGG analyses were conducted for the statistically significant DEGs we identified during the injury and recovery processes. The most dominant terms in the injury phase, both in the overall analysis and in sex-specific subgroups, were “response to xenobiotic stimulus,” “response to lipopolysaccharide,” “response to bacterium/virus,” “inflammatory response,” and “innate immune response.” These findings are consistent with the literature suggesting that gentamicin triggers proinflammatory cytokine production, lipopolysaccharide-like responses, and innate immune pathways (Ali et al. 2011; Althunibat et al. 2022). The data obtained in this study support the observations of previous experimental studies that gentamicin nephrotoxicity involves more pronounced tissue damage in males and a predominant role in immune-inflammatory processes in females (Bennett et al. 1982; Miri et al. 2018). The enrichment of processes such as TNF signaling, antigen presentation, and metabolic adaptation at this stage in males suggests a more comprehensive but relatively homogeneous immune-metabolic response. In contrast, the prominence of processes such as IL-17 and TLR-mediated signaling pathways and negative regulation of viral genome replication in females indicates a more limited but more focused inflammatory response. Regarding sex differences, the predominance of IL-17 and TLR signaling in females is associated with estrogen-mediated antioxidant defense and immune modulation mechanisms. Although NF-κB activity was not directly assessed in the present study, estrogen has been shown in previous experimental models to attenuate NF-κB–dependent transcription, leading to reduced TNF-α expression and a more constrained inflammatory amplification (Evans et al. 2001). In contrast, the enhanced glucocorticoid response, T cell proliferation, and fatty acid β-oxidation observed in males are consistent with the prooxidant and metabolic stress-enhancing effects of testosterone. In males, androgen-associated transcriptional programs and sex-biased epigenetic regulation have been linked to enhanced oxidative stress and sustained inflammatory signaling, potentially explaining the broader injury response observed in this group (Guldan et al. 2024).

Data obtained during the recovery phase indicate that the dedifferentiation, proliferation, and redifferencement processes observed in tubular cells following aminoglycoside toxicity occur at the molecular level. Cell cycle and repair processes driven by sex-specific mechanisms highlight the complex and dynamic nature of the regenerative response. Regulations focused on immune cell migration and metabolic reprogramming in males, and DNA repair and proliferative remodeling in females, provide evidence of the multifaceted control of regeneration. These findings provide molecular support for the regeneration model described in the existing literature (Franco-Acevedo et al. 2021) and reveal that recovery mechanisms differ between sex. The enrichments in the PI3K–Akt, NOD/TLR axis, and fatty acid metabolism observed during the recovery phase are consistent with mitochondrial remodeling and activation of antioxidant genes (e.g., Nrf2, HO-1), suggesting a rebalancing of reparative energy metabolism after gentamicin.

The mechanisms underlying this difference between the sexes may be multilayered. While the antioxidant and anti-inflammatory effects of estrogen explain the more acute but limited inflammatory response in females, the role of testosterone in enhancing renal oxidative stress and the inflammatory response may trigger a more widespread injury response in males (Elsakka et al. 2020). Furthermore, DNMT1 signaling, which is prominent in males, may contribute to a more controlled but longer-lasting recovery pattern in males by mediating differential shaping of the transcriptional program through DNA methylation. Strong TLR/NOD and chemokine signaling in males suggests that immune cell infiltration plays a dominant role, while the increased proliferative DNA repair response in females suggests a faster regenerative capacity of tubular cells. Furthermore, the enhanced fatty acid metabolism and energy adaptation pathways in males suggest a different strategy for compensating for metabolic load during recovery phase. All these findings suggest that the observed differences between the sexes are shaped not only by genetic or environmental factors but also by the interaction of hormonal, epigenetic, immunological, and metabolic regulations.

### Sex-specific cell cycle and repair programs in gentamicin nephrotoxicity

The hub genes identified in this study shed light on the molecular mechanisms of gentamicin-induced nephrotoxicity. The persistent activation of core genes, particularly *CDK1*, *CCNA2*, *BUB1B*, *CDC20*, and *BIRC5*, in both phases (injury and recovery) of the cell cycle and mitotic control pathways underlies the ongoing proliferative stress and unbalanced repair response in renal tubular cells. The literature emphasizes that re-proliferation after injury in tubular epithelial cells is essential for adaptive repair, but that this proliferation, when uncontrolled or dysregulated, can lead to maladaptive repair pathways such as cell cycle arrest, senescence, or fibrosis (De Chiara et al. 2021).

Sex-related hub gene differences, specifically, the male-biased expression of *Nusap1* during the injury phase and *Cenpf* during the recovery phase and the female-specific activation of *Pbk*, *Ccnb1*, and *Nuf2* indicate that gentamicin response is modulated by sex hormones and distinct transcriptional programs. This suggests a tendency for more pronounced mitotic stimulation and associated cell cycle stress in males, while females exhibit a more controlled, but DNA repair-oriented, recovery response. Previous studies have shown that androgens can enhance mitotic activity by increasing the expression of cell cycle genes such as *CDK1* and *CENPF* (Lopes-Ramos et al. 2020; Chen et al. 2025), while estrogen modulates DNA repair pathways (particularly mediated by *PBK* and *CCNB1*) (Kwekel et al. 2013; Lozano-Herrera et al. 2025). Therefore, the male-specific prominence of *Nusap1* (injury phase) and *Cenpf* (recovery phase) may represent stronger mitotic stimulation, whereas the increased expression of Pbk–Ccnb1–Nuf2 in females may represent a more selective, repair-focused response.

Transcription factor analyses also support this picture. *CENPA*, *FOXM1*, and *MYBL2*, prominent in all comparisons, reveal cell cycle reprogramming during gentamicin nephrotoxicity. The role of *CENPA* in maintaining chromatin stability during DNA damage (Zeitlin et al. 2009) and the support of *FOXM1* in proliferative regeneration and antioxidant response (Zhang et al. 2025) highlight the critical role of mitotic regulators in both phases.

*MYBL2*’s G2/M control and cell cycle control via the *p53–CDK1* axis are particularly important for the maintenance of proliferative compensation. *DNMT1*, identified as specific to men, is associated with the restoration of DNA methylation balance through epigenetic regulation. It has been shown that testosterone can suppress the antioxidant response by increasing *DNMT1* activity (Kumar and Brooks 2023). This mechanism is consistent with the more severe oxidative stress and impaired recovery observed in men.

From a pharmacological perspective, these findings highlight the importance of a phase-based approach. The significant upregulation of cell cycle genes during the injury phase suggests that suppressing mitotic stress may be potentially beneficial, leading to the emergence of inhibitors of *BUB1B* (3-phenyl-CPP), *BUB1* (GATX2), *CCNA2* (cordycepin), *CCNB1* (seliciclib), and *BIRC5* (LY2181308) as pharmacological targets. Conversely, activator-type drugs (lubiprostone, niflumic acid) are potentially harmful because they can increase mitotic activity during this phase. Because cellular proliferation is a physiological requirement during the repair phase, mitosis inhibitors require careful evaluation (Humphreys 2018). In this context, recurrent involvement of cell cycle hubs most notably *CCNB1* across both injury and recovery phases highlights their relevance for therapeutic modulation. The sex-dependent emergence of recovery-associated modules, characterized by *PBK–CCNB1–NUF2* in females and *CDCA8* in males, further suggests that regenerative programs are regulated by sex-specific transcriptional networks. These observations support the concept that pharmacological interventions targeting cell cycle–related pathways may require sex and phase-specific optimization rather than uniform suppression. In particular, excessive activation of *BIRC5* has been associated with maladaptive repair and fibrotic progression, indicating that its inhibition may represent a context-dependent therapeutic option during the repair phase (Altieri 2010). From a translational perspective, the functional relevance of *CCNB1* and other cell cycle–associated hubs could be further evaluated using in vitro proximal tubular epithelial cell models or sex-stratified in vivo rodent models, with phase-specific pharmacological modulation. Notably, several inhibitors targeting *CCNB1* and *BIRC5*-related pathways are already under investigation in oncology-focused clinical trials, highlighting the potential for drug repositioning approaches in gentamicin-induced kidney injury.

The targeted analysis of hormone-responsive and repair-associated genes (Table 1) provides additional mechanistic resolution to the sex-dependent transcriptional patterns observed in our study. Several genes included in the targeted expression panel were also independently identified as highly connected hub genes in the PPI network analysis, indicating concordance between expression-based and network-based approaches. The coordinated induction of cell-cycle regulators (*FOXM1*, *MYBL2*, *CENPA*, *CCNB1*, *CCNA2*, *CDK1*, *BUB1*, *BUB1B*) during the injury phase in both sexes is consistent with an acute proliferative response to tubular damage. However, the stronger repression of CENPA and CCNA2 during the recovery phase in males suggests a sex-dependent modulation of mitotic shutdown, which may influence the balance between adaptive and maladaptive repair. Concurrently, the preferential induction of immune and chemokine-related genes (*TLR2*, *TLR4*, *CCL2*, *CXCL10*) in males supports the persistence of inflammatory signaling beyond the injury phase, whereas the expression patterns of *IL1RN* and *SOCS3* indicate a conserved but hormonally modulated interface between estrogen signaling and NF-κB–related inflammatory control.

In addition to this conserved proliferative core, male kidneys exhibited a distinct phase-dependent regulatory pattern involving *DNMT1* and *NR3C1*. *DNMT1* was upregulated during the injury phase and downregulated during recovery, whereas *NR3C1* showed inverse regulation, with reduced expression during injury and increased expression during recovery. This reciprocal pattern suggests a temporal shift from epigenetic remodeling during acute injury toward glucocorticoid-mediated stress regulation during the recovery phase. Together, the convergence of hub gene architecture with phase-specific *DNMT1*–*NR3C1* regulation supports a model in which shared cell-cycle networks are differentially modulated by epigenetic and hormonal control layers in a sex- and phase-dependent manner.

Overall, the findings suggest that phase- and sex-specific pharmacological targets for gentamicin-induced nephrotoxicity differ significantly, and the treatment approach should be optimized not only according to pathological phase but also according to biological sex (Lopez-Novoa et al. 2011).

## Conclusion

This study demonstrates that the injury and recovery phases of gentamicin-induced nephrotoxicity are governed by a common proliferative core in both sexes, but that the responses developing around this core are shaped by sex-specific regulatory networks. Males exhibit a broader but more homogeneous transcriptional reprogramming, while females exhibit a more focused response focused on proliferation and DNA repair.

Hub gene and transcription factor analyses revealed that key cell-cycle regulators such as *CDK1*, *CCNA2*, *BUB1B*, and *CDC20* form a conserved core in both phases, while the female-specific *PBK–CCNB1–NUF2* axis and the male-specific *CDCA8* highlight distinct sex-dependent regulatory modules during the recovery phase. DGIdb screening demonstrated that some of these core genes are pharmacologically targetable, suggesting that suppressing mitotic stress, in particular, may have potential therapeutic value.

Overall, the findings suggest that a phase-based and sex-sensitive approach to the assessment of gentamicin nephrotoxicity provides a more accurate biological framework for understanding pathogenesis and for developing future personalized treatment strategies.

## Supplementary Information

Below is the link to the electronic supplementary material.

ESM 1 Supplementary Material 1: Quality control and differential expression overview for the GSE50804 dataset. (a) Boxplots showing normalized expression distributions across General, Female, and Male subgroups for D15 (injury) and D29 (recovery) phases. (b) UMAP embeddings demonstrating phase- and sex-dependent clustering patterns. (c) Volcano plots displaying significantly up-regulated (red) and down-regulated (blue) DEGs for each comparison (padj < 0.05, |log₂FC| ≥ 1). These QC and DEG overview plots validate normalization consistency, show clear sample clustering across biological groups, and confirm robust differential expression patterns used for downstream GO/KEGG, PPI, TF, and DGIdb analyses. (PNG 787 KB)

ESM 2 Supplementary Material 2: Metadata of all samples used in this study, including GSM accession numbers, treatment conditions, sex, and biological replicates. (File format: Excel; Filename: Supplementary_Table_S1.xlsx) (XLSX 9.64 KB)

ESM 3 Supplementary Material 3: GO Biological Process (BP) and KEGG pathway enrichment results. Includes: Enriched GO-BP terms (FDR < 0.05) Enriched KEGG signaling pathways (FDR < 0.05) Gene counts, enrichment scores, and adjusted p-values. Separate sheets for each group: General, Female, Male (both D15 and D29). These results support the sex-dependent functional interpretation presented in Figs. 2 and 3. File: Supplementary_File_3.xlsx Format: Excel (.xlsx) (XLSX 928 KB)

ESM 4 Supplementary Material 4 (XLSX 182 KB)

ESM 5 Supplementary Material 5: Hub genes, PPI network scores, and transcription factor (TF) rankings. Includes: Top 10 hub genes per group (General/Female/Male × D15/D29) MCC scores from Cytoscape/MCC. TF enrichment results from ChEA3 Sex-specific and phase-specific hub gene lists Candidate gene modules used for DGIdb drug screening This table provides the full datasets underlying Table 1 in the manuscript. File: Supplementary_File_4.xlsx Format: Excel (.xlsx) (XLSX 49.6 KB)

## Data Availability

All datasets analyzed in this study are publicly available from the NCBI-GEO repository (Accession: GSE50804). Processed data, scripts, and analysis outputs are available from the corresponding author upon reasonable request.
