# Supplementary figures and images for: Sex-dependent transcriptional responses and druggable targets in gentamicin-induced nephrotoxicity
Source: Naunyn Schmiedebergs Arch Pharmacol. 2026 Jan 24;399(7):9817–29. doi: 10.1007/s00210-026-04997-4 (PMC13152955; doi:10.1007/s00210-026-04997-4)

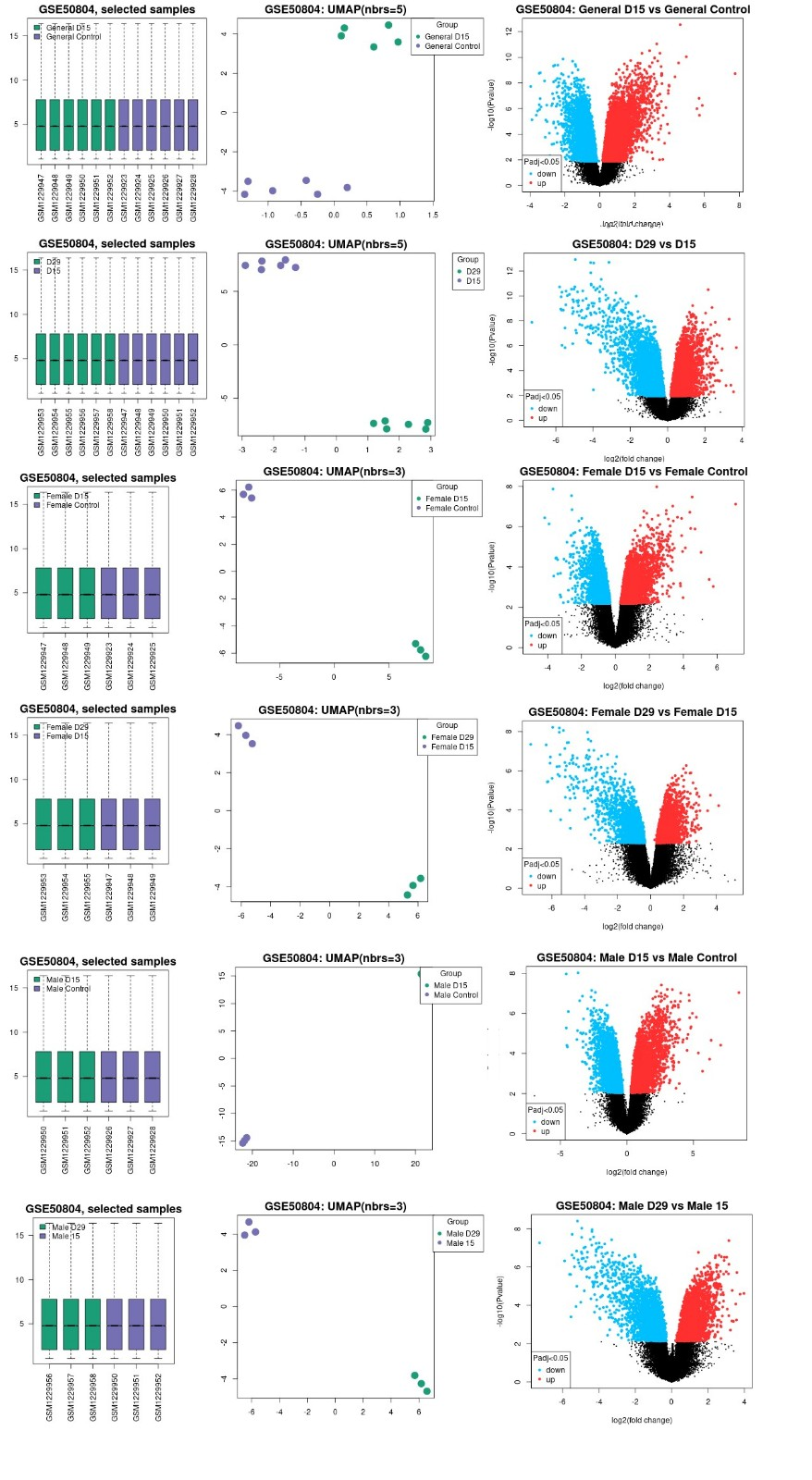

Supplement: Supplementary file 1 — Supplementary Material 1: Quality control and differential expression overview for the GSE50804 dataset. (a) Boxplots showing normalized expression distributions across General, Female, and Male subgroups for D15 (injury) and D29 (recovery) phases. (b) UMAP embeddings demonstrating phase- and sex-dependent clustering patterns. (c) Volcano plots displaying significantly up-regulated (red) and down-regulated (blue) DEGs for each comparison (padj < 0.05, |log₂FC| ≥ 1). These QC and DEG overview plots validate normalization consistency, show clear sample clustering across biological groups, and confirm robust differential expression patterns used for downstream GO/KEGG, PPI, TF, and DGIdb analyses. (PNG 787 KB) [file 210_2026_4997_MOESM1_ESM.png]
